# Artificial Intelligence in the Detection of Papilledema: A Systematic Review

**DOI:** 10.3390/jcm15134878

**Published:** 2026-06-23

**Authors:** Ovidiu Samoilă, Vasiliki Antonopoulou, Lăcrămioara Samoilă

**Affiliations:** 1 Department of Ophthalmology, “Iuliu Hațieganu” University of Medicine and Pharmacy, 400012 Cluj-Napoca, Romania; ciprian.samoila@umfcluj.ro; 2 Faculty of Medicine, “Iuliu Hațieganu” University of Medicine and Pharmacy, 400012 Cluj-Napoca, Romania; vasiliki.antonopoulou000@gmail.com; 3 Department of Physiology, “Iuliu Hațieganu” University of Medicine and Pharmacy, 400012 Cluj-Napoca, Romania

**Keywords:** artificial intelligence, optic disc, papilledema, deep learning, fundus imaging

## Abstract

**Background/Objectives**: This review explores the role of artificial intelligence (AI), particularly with deep learning and machine learning, in the detection and classification of papilledema using retinal fundus imaging. **Methods**: The study synthesizes historical, technical, and clinical insights, comparing AI-based diagnostic accuracy with conventional methods. **Results**: Our findings demonstrate that AI systems, especially convolutional neural networks (CNNs), offer sensitivity and specificity comparable to, or even surpassing, expert-level fundoscopy. **Conclusions**: These results suggest significant implications for early diagnosis, triage, and telemedicine integration in ophthalmic care.

## 1. Introduction

Papilledema refers to optic disc swelling due to elevated intracranial pressure (ICP), often signalling serious conditions, such as brain tumours, hydrocephalus, meningitis, or venous sinus thrombosis [1]. Early stages may be asymptomatic or present with transient visual disturbances and headaches, requiring prompt differentiation from benign mimics like pseudopapilledema. Papilledema may signify a life-threatening disease, and early detection could save lives. 

Diagnosis of papilledema typically involves fundoscopic examination to visualize optic nerve swelling [2], though accuracy depends on examiner expertise and pupil dilatation with mydriatic drops [3]. Traditional fundoscopy lacks a standardized recordable format, limiting reproducibility and utility for AI training [4].

In contrast, retina photography is easily performed, even through non-mydriatic pupils, and expert-graded fundus photographs provide structured, consistent data. Various non-mydriatic fundus cameras exist on the market, and photos can be captured in seconds. AI models trained on such images can detect subtle diagnostic features with high precision, offering potential advantages in accuracy and accessibility, particularly where specialist availability is limited.

### 1.1. Historical Background

The term artificial intelligence (AI) was first coined in 1955 [5], but foundational ideas date back to Alan Turing’s work in the 1940s, including the Turing Test [6]. The field gained momentum during the “Golden Age of AI” (1956–1974), which saw the rise in early neural networks [6]. Since 2020, advances in algorithms have led AI systems to outperform humans in image recognition tasks [7].

In ophthalmology, AI was first explored in the 1970s. A notable breakthrough came with models capable of identifying optic disc and fovea with over 99% accuracy. The FDA-approved iDx-DR system marked a major milestone for AI in clinical diagnostics [8].

The rise in deep learning (DL), especially with convolutional neural networks (CNNs) and the launch of ImageNet in 2009, further propelled AI in ophthalmology [9,10]. DL models now aid in diagnosing conditions, like diabetic retinopathy, glaucoma, and macular diseases, and are integral to future clinical decision-making, particularly due to ophthalmology’s image-heavy nature.

### 1.2. Fundamentals of AI, ML, and DL

Artificial intelligence through machine learning (ML) enables computers to learn from data via a process known as inferencing [11]. There are two primary learning approaches: supervised, which uses labelled data (e.g., images marked as diseased or healthy), and unsupervised, which detects patterns from unlabelled data by grouping similar features. Both methods typically require input from a domain expert to translate raw data, like pixel values, into meaningful feature vectors. Deep learning, a subset of ML, eliminates this dependency by automatically extracting features from unprocessed inputs. Using multiple neural layers, DL transforms raw data into increasingly abstract representations, allowing for the detection of highly complex patterns that may exceed human interpretability.

In supervised learning, the machine is first trained using a series of input–output pairings in order to learn to recognize a specific outcome when given an input. This pattern of associations is treated as knowledge, which can then be generalized to predict outputs for new unseen inputs [12]. Supervised learning forms the basis of many artificial intelligence applications and relies on large sets of labelled data. Common supervised learning algorithms include the Naïve Bayes method, support vector machines (SVMs), Decision Trees, and Artificial Neural Networks (ANNs). These approaches are especially useful when the goal is classification or regression based on clearly defined inputs and known outputs [13]. One of the most widely known supervised learning methods is K-Nearest Neighbours (KNN), which classifies input data by measuring similarity to labelled examples in the training dataset. Although primarily a supervised algorithm, KNN has also been adapted for semi-supervised learning applications [14].

Unsupervised learning, on the other hand, is employed when labelled outputs are unavailable. It uses only the input features to discover hidden structures, distributions, or groupings within the data. The objective is to allow the algorithm to explore and segment the input space without prior guidance on what the output should be. In medical imaging, unsupervised learning is frequently used to divide a set of pixels into regions of interest versus background or to identify patterns across homogeneous groups of data.

Deep learning has been particularly transformative in the field of ophthalmology. Deep learning techniques typically used in this field fall into three broad categories: pre-trained unsupervised networks (PUNs), convolutional neural networks (CNNs) and traditional neural networks (NNs) [15,16]. Among these, CNNs are the most widely used and have shown exceptional performance in the analysis of medical images. Multilayer Perceptrons (MLPs), also known as Deep Feedforward Networks (DFNs), are another category of deep learning models. These networks aim to approximate a target function by defining a mapping between input and output variables, using adjustable weights that are re-refined through training on data [17].

For two-dimensional data, such as digital retinal images, convolutional neural networks are particularly well-suited [16,18]. They automatically extract features from the raw image and then combine semantically related patterns to build hierarchical representations. This feature extraction process helps reduce redundancy or sparsity in the image data, preserving critical diagnostic information. CNNs typically operate in multiple stages, each consisting of convolutional layers, non-linear activation layers, and pooling layers. The early stages focus on mapping fundamental image features, while deeper stages combine these features to form increasingly abstract representations. The depth of a CNN refers to the number of layers in the network, and its width is determined by the number of filters or neurons used at each stage.

The final stage of a CNN typically consists of one or more fully connected layers, which function similarly to traditional neural networks by integrating the high-level features extracted in earlier layers and generating the final classification output (e.g., presence or absence of disease). A key component of CNN architecture is the pooling operation, which aggregates spatially adjacent features and reduces the dimensionality of the data. Common approaches include max pooling, in which only the maximum value within a local region is retained. This process emphasizes the most salient features while reducing computational complexity and improving robustness to minor variations such as shifts or distortions in the input images. Overall, deep learning—particularly CNN-based approaches—has become central to medical image analysis, offering high accuracy and efficiency in ophthalmic diagnostics [16,17].

### 1.3. AI Applications in Ophthalmology

Artificial intelligence has significantly advanced the field of ophthalmology, particularly through DL systems like Retinal AI Diagnosis System (RAIDS), which can detect up to ten retinal diseases from fundus photographs [19]. Modern DL models, including CNNs and Vision Transformers, have achieved exceptional accuracy up to 99.17% in screening for retinal conditions, including hereditary retinal disorders (IRDs) such as retinitis pigmentosa and Stargadt’s disease [20,21]. These models perform tasks like segmentation, classification, and prediction with high precision.

Innovative frameworks, such as those based on VGG19 (developed by Visual Geometry Group at the University of Oxford) with transfer learning from ImageNet, have further enhanced diagnostic capabilities [22,23]. Other notable developments include algorithms for detecting age-related macular degeneration (AMD), retinopathy of prematurity (ROP), and keratoconus, each with diagnostic accuracy exceeding 90% [24,25]. Beyond retinal diseases, similar architectures have been applied to corneal and ocular surface conditions, such as keratoconus and meibomian gland dysfunction (MGD). In meibography-based analysis, deep learning systems have demonstrated diagnostic performance with AUC values around 0.90, as well as sensitivity and specificity commonly reported in the range of 85–92%, depending on dataset characteristics and imaging protocols [26].

In addition to deep learning approaches, machine learning (ML) methods—including supervised and unsupervised learning—have also demonstrated value in ophthalmology. Supervised learning algorithms are trained on labelled datasets to perform classification or regression tasks, whereas unsupervised learning identifies latent structures within unlabelled data and is particularly useful for high-dimensional datasets, such as corneal topography and imaging-based phenotyping [27,28]. Reinforcement learning, which optimizes decision-making through trial-and-error feedback and reward-based systems, is an emerging approach being explored for applications such as surgical planning, treatment optimization, and adaptive image analysis [29]. Collectively, these artificial intelligence techniques are increasingly contributing to improvements in ophthalmic diagnosis, treatment planning, and clinical outcome prediction.

This review aims to systematically evaluate the role of artificial intelligence (AI) in the detection, classification, and severity assessment of papilledema. Given the potentially life-threatening conditions associated with elevated intracranial pressure, timely recognition of papilledema is of particular clinical importance. We therefore sought to assess the diagnostic performance of machine learning and deep learning approaches developed specifically for papilledema detection and grading. Given the rapid evolution of AI technologies, we seek to systematically assess and compare the performance of various machine learning (ML) and deep learning (DL) approaches applied to this condition. Furthermore, this review evaluates the current level of evidence supporting these methods and examines their readiness for integration into routine clinical practice, with particular attention to diagnostic accuracy, reproducibility, and real-world applicability.

## 2. Materials and Methods

### 2.1. Study Design

This is a systematic review, based on a comprehensive literature search conducted across databases including PubMed, Cochrane, Clinicaltrials.gov, and Google Scholar to identify research involving artificial intelligence (AI) in the detection of papilledema through fundoscopic examination. Search terms combined keywords related to “artificial intelligence”, “papilledema”, and “fundus imaging” or “retinal photography”.

The inclusion criteria encompassed studies involving patients of any age with suspected or confirmed papilledema; the use of AI-based diagnostic tools, such as machine learning or deep learning algorithms; and comparisons to standard fundoscopic examination. Eligible studies were required to report diagnostic performance metrics, like sensitivity, specificity, or area under the curve (AUC). Only studies in which papilledema detection, classification, or severity assessment constituted a primary outcome were eligible for inclusion. Acceptable study designs included randomized controlled trial cohorts and case–control and cross-sectional studies, with restrictions on publication date (2016–2025).

Exclusion criteria ruled out animal studies, investigations not involving AI, studies unrelated to papilledema, reviews, editorials, case reports with fewer than 10 participants, and studies lacking relevant outcome measures or proper comparisons. Studies focused primarily on general optic disc abnormality classification, optic neuropathies, optic disc drusen, pseudopapilledema differentiation without dedicated papilledema outcomes, OCT-based structural analysis without fundus photograph-based papilledema assessment, or multimodal optic nerve disease classification were excluded. Non-English studies were excluded unless translation was possible. After applying these criteria, duplicate and irrelevant articles were removed. Titles and abstracts were screened, and full texts were reviewed for final eligibility.

Artificial intelligence tools were used exclusively to assist with language editing, grammar refinement, and improvement of manuscript readability. All literature searches, study selection, data extraction, quality assessment (QUADAS-2), interpretation of findings, preparation of tables, and formulation of the final scientific conclusions were performed independently by the authors. All figures, with the exception of Figure 5, were created independently by the authors. Figure 5 was generated with the assistance of AI-based tools and subsequently reviewed and modified by the authors. All AI-assisted text modifications were carefully reviewed and verified by the authors, who assume full responsibility for the accuracy, integrity, and scientific content of the manuscript. The review was prepared following PRISMA guidelines and was registered on OSF Registries platform (artificialintelig—https://osf.io/aj7ds/overview?view_only=d94db839bfa04a99b012624002d4e7e9 (accessed on 1 June 2026)). The completed PRISMA checklist is provided in the Supplementary Materials (Supplementary Material File S1).

### 2.2. Outcomes

Eight studies met the inclusion criteria and were selected for further analysis. Key data extracted from each study included publication year, sample characteristics, methodology, follow-up duration, interventions, and diagnostic outcomes.

The primary outcome was the diagnostic accuracy of AI models in identifying and grading papilledema. Measures such as sensitivity, specificity, and AUC were used with confidence intervals reported where available. In cases where comparisons with expert evaluations were made, relative risks and odds ratios were also analyzed.

### 2.3. Statistic Analysis and Bias

The results were analyzed using appropriate statistical tools to ensure robust evaluation of AI performance in clinical settings. The Quality Assessment of Diagnostic Accuracy Studies-2 (QUADAS-2) [30] assessment method was conducted to evaluate the methodological quality and potential bias and to determine how reliable and valid the results of diagnostic accuracy studies are.

## 3. Results

### 3.1. Literature Research

**Figure 1:**
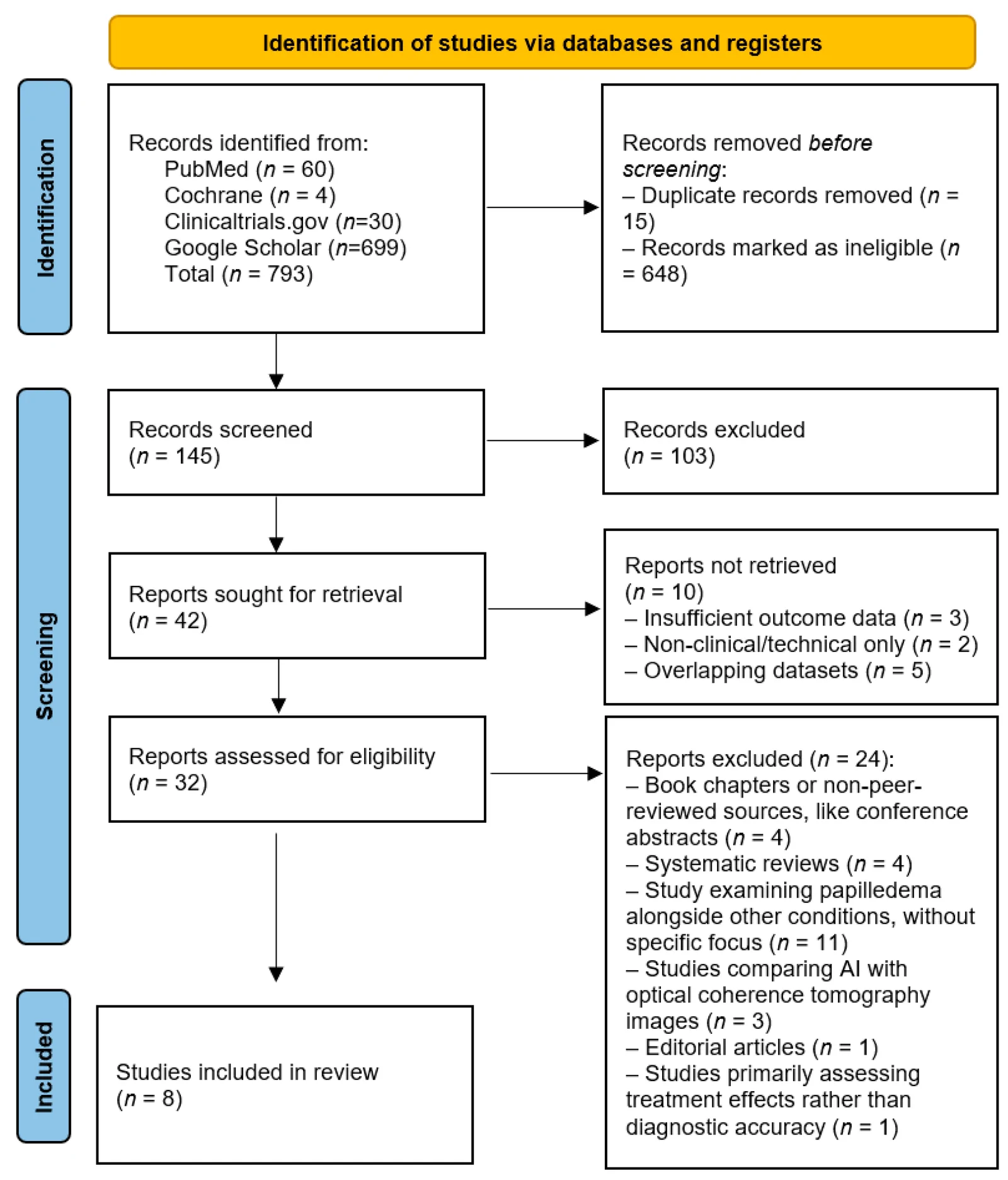
PRISMA flow diagram; 8 studies met the inclusion criteria.

The study selection process began with a systematic search across major databases, which yielded 145 initial records related to the application of artificial intelligence (AI) for detecting papilledema from ocular fundus photographs (Figure 1).

After initial screening, 43 studies were shortlisted for full-text assessment. Following an initial screening process, 10 studies were not available and 24 studies were excluded for the following reasons: book publications (*n* = 3), conference abstracts (*n* = 1), systematic reviews (*n* = 4), studies analyzing papilledema in conjunction with other conditions without clear differentiation (*n* = 11), comparisons between AI and optical coherence tomography (OCT) images rather than human experts (*n* = 3), editorial articles (*n* = 1), and studies primarily assessing treatment effects rather than diagnostic accuracy (*n* = 1). The excluded studies and reasons for exclusion are summarized in Table 1, with further detail provided on the methodological grounds for exclusion, such as lack of specificity to papilledema or reliance on non-fundoscopic imaging. Many of the excluded studies addressed broader optic disc abnormalities or pseudopapilledema rather than papilledema as a primary diagnostic target.

Ultimately, eight studies were selected for final inclusion, all of which evaluated the diagnostic accuracy and clinical utility of AI models compared with expert neuro-ophthalmologists (Table 2).

The included studies exhibited considerable diversity in their AI techniques, validation strategies, datasets, and reported outcomes. Among the selected works, various AI architectures were used, ranging from traditional machine learning approaches such as support vector machines (SVMs) to advanced deep learning models like DenseNet, U-Net, and the BONSAI (Brain and Optic Nerve Study with Artificial Intelligence) deep learning system. Dataset sizes varied significantly, from 100 to over 15,000 fundus images, with only three studies, namely the studies by Milea et al. [61], Chang et al. [56], and Lin et al. [57], incorporating multi-centre external validation. These differences allowed for the broad assessment of AI applicability and performance across varied clinical environments and patient populations.

### 3.2. Study Analysis

Each study demonstrated promising results regarding AI’s capability to detect and classify papilledema accurately. Akbar et al. [62] employed an SVM model and achieved 92.86% accuracy, while Milea et al. [61] reported a near-perfect AUC of 0.99 using DenseNet and U-Net on a large, multiethnic dataset. Vasseneix et al. [60] applied the BONSAI model to grade papilledema severity and achieved high diagnostic accuracy (AUC 0.93). Saba et al. [59] also demonstrated remarkably high precision (accuracy 99.17%) through vessel segmentation using combined DenseNet and U-Net. In more practical settings, Azarmina et al. [58] showed 85% agreement between a computer-aided diagnosis (CAD) system and clinicians, suggesting real-world feasibility. Lin et al. [57] validated the BONSAI system for pediatric use with excellent results (AUC 0.99, sensitivity 98.0%, specificity 94.1%), and Biousse et al. [55] demonstrated the utility of AI-assisted diagnosis in emergency departments, achieving an AUC of 0.97, sensitivity of 84.0%, and specificity of 98.9%. Chang et al. [56] used a DenseNet-based tri-branch CNN to differentiate pediatric true papilledema from pseudopapilledema. They reported an AUC of 0.81 on the external test set and sensitivity of 90.4%, with higher sensitivity than expert pediatric neuro-ophthalmologists.

All the above-mentioned study characteristics are further categorized and summarized in Table 3.

Sensitivity across studies ranged from 84.0% to 98.0%, specificity reached 98.9%, and overall accuracy reached 99.17% in selected studies. These results often matched or exceeded expert-level performance. In comparative analysis, deep learning models, particularly DenseNet, U-Net, and BONSAI, consistently outperformed traditional machine learning systems, such as SVM and CAD. Deep learning models showed higher AUC scores and better sensitivity/specificity metrics, confirming their superiority in papilledema diagnostics. The overall findings indicate that AI not only improves diagnostic accuracy but also enhances patient triage and management strategies, solidifying its emerging role in modern neuro-ophthalmological practice. The studies included in this systematic review demonstrated the continuous evolution of AI models in papilledema detection, ranging from early machine learning approaches such as SVMs [62] to more sophisticated deep learning algorithms like DenseNet and U-Net [56,59,61]. The datasets varied significantly in size from small-scale investigations to large multiethnic cohorts, ensuring sturdy external validation.

In addition to differences in model architecture, substantial variability was observed in training and validation strategies across the included studies. Earlier investigations, such as those by Akbar et al. [62] and Saba et al. [59], primarily relied on internal validation procedures, including cross-validation or expert-panel comparison, without independent external testing. In contrast, more recent studies increasingly incorporated multi-centre datasets and external validation cohorts to improve model generalizability. Chang et al. [56] combined 10-fold cross-validation with an independent external test set, while Lin et al. [57] and Milea et al. [61] validated their models across multiple centres and diverse patient populations. Similarly, Biousse et al. [55] evaluated a previously developed deep learning system in a prospective real-world emergency department setting. This evolution from internally validated models toward externally validated, multi-centre studies reflects a growing emphasis on clinical applicability and robustness, while also highlighting persistent heterogeneity in methodological design that should be considered when comparing reported diagnostic performance across studies.

A major advancement highlighted by the studies is the transition of AI applications from static laboratory-based evaluations to real-world settings, such as emergency department and telemedicine platforms. Studies like those by Biousse et al. [55] showcase AI’s potential in real-time triage and diagnostic decision-making, reducing dependency on specialist availability. Similarly, Chang et al. [56] and Lin et al. [57] bring forth critical insights into the use of AI for younger patients (pediatric population), a demographic where papilledema detection is particularly challenging due to differential diagnoses such as pseudopapilledema.

Additionally, models, particularly those in Vasseneix et al. [60] and Saba et al. [59], have explored AI’s potential for determining treatment urgency. The ability of AI models to distinguish between mild, moderate, and severe papilledema provides valuable clinical insights, ensuring that high-risk patients receive appropriated medical intervention in a prompt and timely manner.

All in all, the studies included in our systematic review provide compelling evidence that AI can match or even exceed human expert performance in papilledema detection and classification. DenseNet, U-Net, and BONSAI [55,56,57,59,60,61] demonstrated higher accuracy compared to traditional machine learning approaches (SVM, CAD) [58,62].

### 3.3. Quality of Evidence

**Figure 2:**
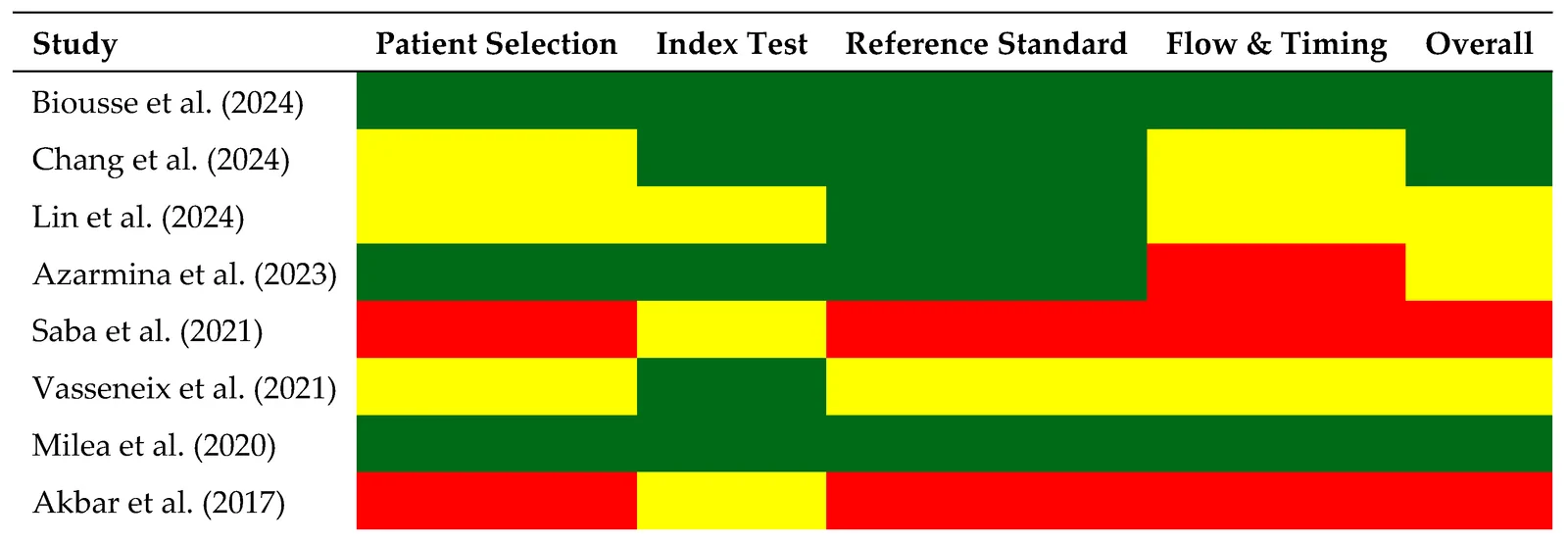
Traffic light matrix of QUADAS-2 assessment results. Green—low risk; yellow—moderate risk; red—high risk. Based on references [56,57,58,59,60,61,62,63].

The QUADAS-2 assessment method was used to evaluate the methodological quality and potential bias of the eight studies included in this systematic review. The analysis focuses on key domains including patient selection bias, index test bias (AI model), reference standard bias, flow and timing bias, and applicability concerns (Table 4, Figure 2).

1. Patient Selection Bias (evaluation of the study populations, concerning its appropriate selection and representation). Studies by Biousse et al. [55], Azarmina et al. [58], and Milea et al. [61] exhibited low risk of bias in patient selection. The datasets used were appropriately designed for AI-based analysis, ensuring that they included a diverse range of patients and fundus image sources and thus contributing to the broad applicability of the AI models assessed. On the other hand, Saba et al. [59] and Akbar et al. [62] demonstrated a high risk of bias in the patient selection domain due to the use of relatively small and potentially non-representative datasets, as well as limited reporting regarding patient recruitment methodology. In both studies, image selection appeared to rely on retrospective or convenience sampling approaches rather than consecutive or randomized inclusion, increasing the likelihood of selection bias. Furthermore, the datasets lacked broad demographic and clinical heterogeneity, reducing confidence in the generalizability of the models to real-world populations. These limitations are particularly relevant in papilledema research, where optic disc appearance may vary substantially depending on disease severity, patient age, ethnicity, image acquisition conditions, and the presence of confounding optic nerve abnormalities.
2. Index Test Bias (for bias in the AI model’s application and interpretation). Most studies showed a low risk of bias in the application of AI algorithms. However, Akbar et al.’s [62] study resulted in a moderate risk due to the use of an SVM model without extensive external validation. In contrast, the studies by Milea et al. [61], Chang et al. [56], and Lin et al. [57] demonstrated strong methodological precision, focusing on external multi-centre validation to minimize bias.
3. Reference Standard Bias (examines the accuracy and consistency of the reference group of experts). The reference standard in most studies was expert neuro-ophthalmologists, ensuring a high level of reliability. However, Saba et al. [59] and Akbar et al. [62] were assessed to have a high risk of bias in this section because of the inconsistencies in human grading methods, leading to probable variability in the reference standard.
4. Flow and Timing Bias. While Biousse et al. [55] and Milea et al. [61] showed low risk, Azarmina et al. [58], Saba et al. [59], and Akbar et al. [62] were considered as high risk studies due to potential discrepancies in timing between AI–human comparisons. Differences in imaging time points and delays in manual assessment could introduce variations that affect the validation models.

Based on the above-mentioned domains in quality assessment of studies, we conclude the three categories for assessment of risk (low, moderate and severe risk) and rank the studies as such:

- Low-risk studies: The studies by Milea et al. [61], Chang et al. [56], and Biousse et al. [55] had the lowest risk in all domains evaluated, which strengthens the confidence in their results for future applications. The study by Chang et al. [56] demonstrated overall low risk of bias across most QUADAS-2 domains, with unclear risks in patient selection and flow due to its retrospective design (very common in AI studies), while maintaining low applicability concerns. However, the study is methodologically strong because of multi-centre applications and external validation. Biousse et al. [55] is one of the strongest studies, as a real-world design and use of non-mydriatic fundus photos in ED setting.
- Moderate-risk studies: The studies by Azarmina et al. [58], Vasseneix et al. [60], and Lin et al. [57] showcase moderate concerns in reference standards and patient selection, that indicated the existence of limitations in how the AI models were trained and validated.
- High-risk studies: The studies by Akbar et al. [62] and Saba et al. [59] demonstrated elevated risk, especially in regard to patient selection and reference standard, since they carried out research on a limited number of patients, in single centres, and with minimal external validation.

## 4. Discussion

### 4.1. Principal Findings

This systematic review reinforces the growing significance of artificial intelligence (AI) in the detection and classification of papilledema, highlighting its substantial potential to transform neuro-ophthalmological diagnostics. Unlike broader reviews of optic disc abnormalities, the present study specifically focused on papilledema because of its direct association with elevated intracranial pressure and the potential need for urgent neurological evaluation.

Across the included studies, deep learning architectures, such as DenseNet, U-Net, and BONSAI, consistently demonstrated high diagnostic performance. Reported sensitivity values ranged from 84.0% to 98%, while area under the curve (AUC) scores reached as high as 0.99, indicating excellent discriminatory ability. Overall accuracy reached 99.17% in selected studies.

Notably, several studies demonstrated that AI systems were able to match or even surpass the diagnostic performance of expert neuro-ophthalmologists, particularly in the detection and grading of papilledema severity. For example, Milea et al. [61] reported an AUC of 0.99, representing one of the highest performances among the studies included. In addition to detection, multiple AI models showed strong capability in stratifying disease severity, a clinically critical function that directly impacts decision-making and urgency of intervention. These findings collectively suggest that AI systems are capable of not only identifying papilledema but also supporting nuanced clinical judgments that are traditionally reliant on specialist expertise.

Importantly, these results indicate that AI has the potential to serve as a reliable diagnostic adjunct. By reducing diagnostic variability and minimizing the risk of misclassification, AI systems may enhance consistency in clinical practice. Furthermore, their ability to provide rapid analysis positions them as valuable tools for expediting triage, particularly in urgent or resource-constrained settings.

### 4.2. Clinical Implications

Papilledema is the swelling of the optic disc secondary to increased intracranial pressure and may occur in a wide range of conditions, including intracranial tumours, meningitis, cerebrospinal fluid (CSF) disorders, or drug-related causes. Conventional diagnosis relies on expert evaluation, namely ophthalmoscopy, with direct visualization of the retina through a dilated pupil using an ophthalmoscope or fundus photography, whereas standard images are typically obtained after pharmacological mydriasis. The differential diagnosis includes other causes of optic disc edema, such as optic neuritis (inflammatory, ischemic, or toxic) and pseudopapilledema, most commonly due to optic disc drusen.

**Figure 3:**
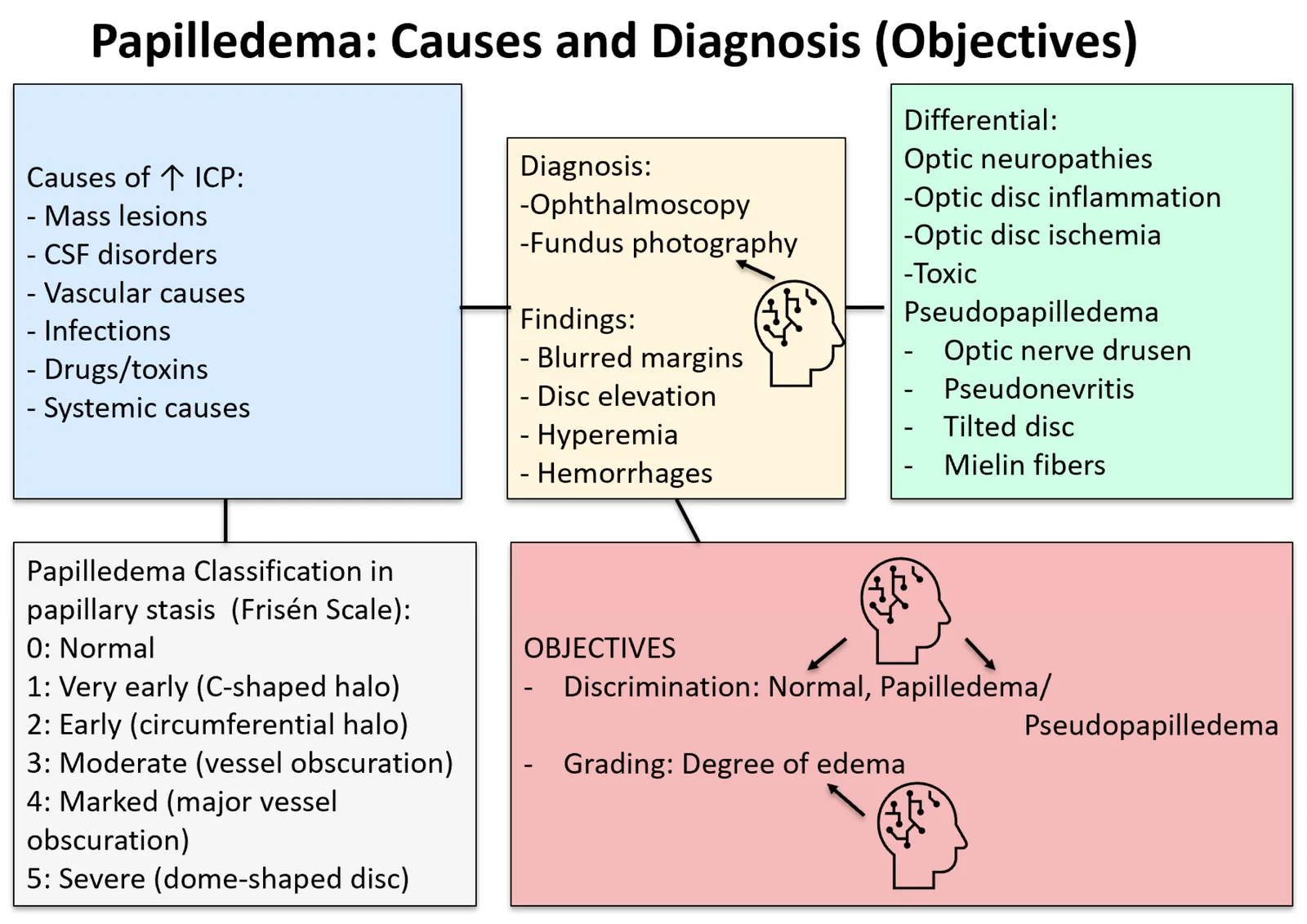
Papilledema—causes and diagnosis (including differential). Frisén scale of papilledema is also presented. AI implementations in diagnosis and the objectives of discrimination are highlighted.

AI implementation is relevant to fundus photography. It could allow the differentiation of papilledema from pseudopapilledema or even the grading of papilledema. Figure 3 highlights the causes of papilledema alongside AI integration with the objectives of diagnosis (detection and grading, including Frisén grading of papilledema).

**Figure 4:**
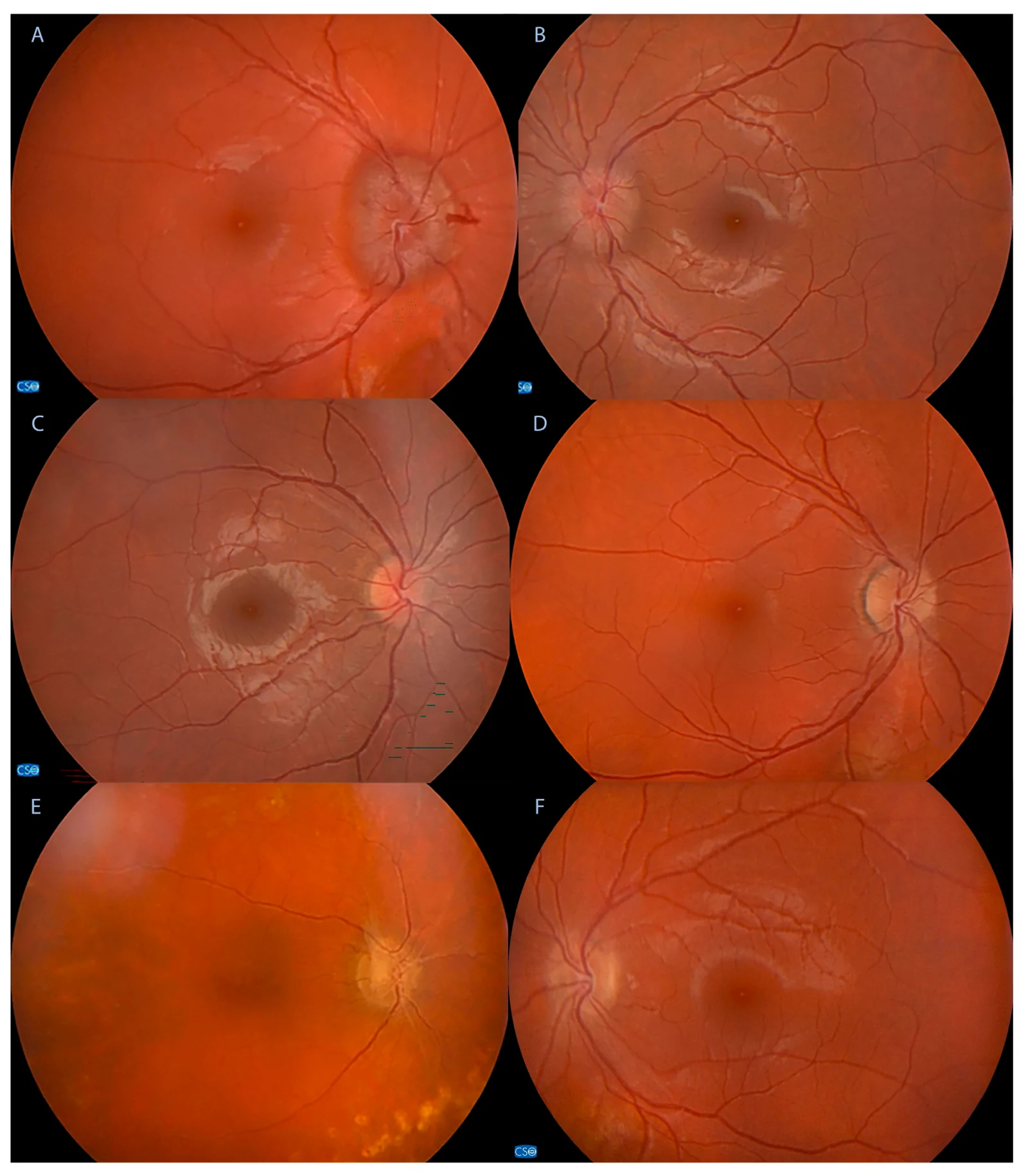
Optic disc edema, with multiple aspects and differential diagnosis (fundus photography, CSO^®^ Fundus Camera). (**A**)—Papilledema Frisén grade 5 and peripapillary hemorrhage; (**B**)—papilledema Frisén grade 3; (**C**,**D**)—papilledema Frisén grade 1; (**E**)—pseudoedema (prepapillary membrane in diabetic retinopathy); (**F**)—pseudoedema (tilted disc) (images derived from personal database, author O.S., unpublished).

Differential diagnosis based solely on fundus imaging remains challenging, sometimes even for experienced clinicians. Optic disc edema is a nonspecific finding, and papilledema represents only one of its potential causes. As illustrated in Figure 4, the presentation of papilledema is heterogeneous and extends beyond blurred disc margins and elevation. Additional ophthalmoscopic features, including retinal hemorrhages and associated retinal abnormalities, should be considered to improve diagnostic accuracy.

The distinction between papilledema and other causes of optic disc swelling remains a major diagnostic challenge. Although some included studies evaluated differentiation from pseudopapilledema, the primary focus of this review remained AI systems developed for papilledema detection and severity assessment.

Among the eight studies, four studies clearly addressed papilledema grading or severity assessment: Azarmina et al. [58], Saba et al. [59], Vasseneix et al. [60], and Akbar et al. [62]. The strongest and most clinically relevant grading evidence comes from Vasseneix et al. [60], which specifically evaluated deep learning-based classification of papilledema severity, and reported performance comparable to neuro-ophthalmologists. Table 5 synthesizes the clinical application of AI in the reviewed studies regarding severity assessment.

In pediatric populations, where diagnostic challenges are particularly pronounced, AI also shows considerable promise. Lin et al. [57] and Chang et al. [56] highlighted the role of AI in differentiating papilledema from pseudopapilledema, a distinction that is often difficult even for experienced clinicians. Accurate differentiation is crucial, as misdiagnosis may lead to unnecessary invasive procedures, such as lumbar puncture. By improving diagnostic confidence, AI systems could reduce patient burden and enhance safety.

From a clinical perspective, the studies included in this review demonstrate that AI applications are highly versatile and adaptable across diverse healthcare environments. AI-assisted detection of papilledema has been evaluated in settings ranging from tertiary referral centres to telemedicine platforms and emergency departments (ED) [55].

One of the most promising applications is emergency triage. Rapid identification of papilledema is critical, as it may indicate elevated intracranial pressure requiring urgent intervention. The study by Biousse et al. [55] demonstrated that AI-assisted triage systems can significantly improve efficiency by rapidly analyzing fundus images and prioritizing high-risk patients. This approach has the potential to reduce unnecessary neuroimaging while ensuring that patients requiring urgent evaluation are promptly identified. The study also demonstrated that non-mydriatic fundus photography can be effectively utilized without compromising the accuracy of papilledema detection. This approach is particularly well suited to the demanding conditions of emergency departments, where time constraints often preclude pharmacological pupil dilation.

AI also has significant implications for telemedicine and underserved regions. In areas with limited access to neuro-ophthalmologists, AI tools can facilitate early detection and appropriate referral by enabling non-specialists to perform initial screening. This capability is particularly relevant in low-resource settings, where delays in diagnosis can lead to adverse outcomes. Additionally, AI may reduce reliance on advanced imaging modalities by flagging cases that genuinely require further investigation, thereby optimizing resource allocation.

**Figure 5:**
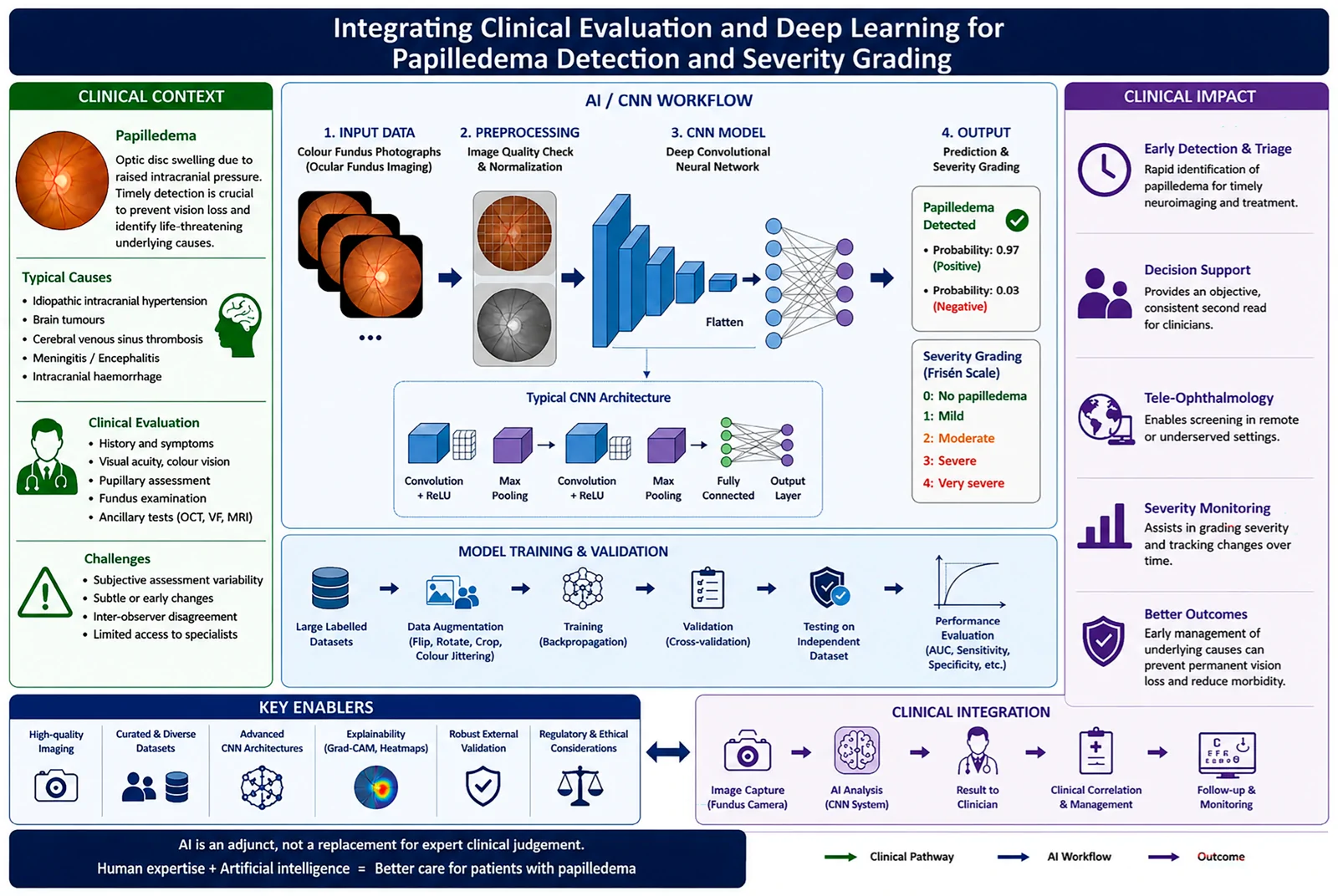
Schematic representation of the integration of clinical evaluation and deep learning-based analysis for papilledema detection. Figure created using AI-assisted tools.

Overall, these applications underscore AI’s potential to enhance clinical decision-making, improve workflow efficiency, and expand access to high-quality diagnostic care. Figure 5 illustrates the possibilities of clinical integration of AI and deep learning for the detection and severity grading of papilledema.

Digital image analysis constitutes a core component of artificial intelligence (AI)-based systems for retinal fundus screening and diagnosis, particularly in the automated detection of optic disc abnormalities, such as papilledema [55,60,61]. Within these pipelines, preprocessing plays a critical role in enhancing image quality and improving feature representation prior to model training and inference. Common preprocessing techniques include intensity normalization, contrast enhancement, and noise reduction, which facilitate the detection of vascular patterns and pathological features relevant for automated analysis [58,59,60,61].

Although not always explicitly reported, preprocessing steps, such as image standardization and optic disc localization, are essential in ensuring robustness across heterogeneous datasets. This is particularly relevant in multi-centre and real-world settings, as demonstrated in the studies by Milea et al. [61], Biousse et al. [55], and Chang et al. [56], where variability in image acquisition required consistent preprocessing to maintain diagnostic performance.

From a computational perspective, fundus images are treated as discrete pixel matrices, enabling efficient numerical processing and allowing deep learning models to extract spatial and textural features relevant for diagnosis [62]. In most studies, colour fundus images are processed in standard RGB format, where each image is represented as a three-channel input corresponding to red, green, and blue intensities. While additional image enhancement or transformation techniques may be applied, these are variably reported across the included studies [58,59].

A representative example is the study by Akbar et al. [62], where retinal fundus photographs underwent preprocessing and feature extraction before classification by a support vector machine (SVM). Instead of analyzing raw images directly, the algorithm relied on preprocessing techniques designed to enhance relevant anatomical structures and facilitate extraction of papilledema-associated image features. This approach illustrates the importance of image standardization, vessel segmentation, optic disc localization, and feature engineering in traditional machine learning systems. Standardized preprocessing pipelines may improve reproducibility and facilitate comparison across studies. Following preprocessing, images are analyzed during the inference stage, where AI models perform tasks, such as classification or severity grading. Convolutional neural networks (CNNs) represent the predominant architectures used in this context, owing to their ability to automatically learn hierarchical feature representations from pixel-level data. Their effectiveness has been demonstrated across multiple studies, achieving high diagnostic accuracy in both detection and grading of papilledema from fundus photographs [55,59,60,61].

Beyond preprocessing, several methodological factors may influence AI performance but were variably reported across the included studies. Data augmentation techniques, such as image rotation, flipping, scaling, cropping, and brightness adjustment, are commonly employed in deep learning workflows to increase dataset diversity and reduce overfitting. Similarly, the quality of dataset annotation remains a critical determinant of model accuracy, as most studies relied on expert neuro-ophthalmologist grading as the reference standard. Differences in annotation protocols, disease classification criteria, and image labelling strategies may contribute to variability in reported outcomes. Future studies would benefit from greater standardization of preprocessing pipelines, annotation procedures, and validation methodologies to improve reproducibility and facilitate direct comparison between AI systems developed for papilledema detection and severity assessment.

This AI workflow is also presented in Figure 5. AI is an adjunct to and not a replacement for expert clinical judgement. Combining AI and human expertise means better care for patients, including those with papilledema. Better outcomes are provided by early management of underlying causes that could prevent vision loss and morbidity.

Despite excellent diagnostic performance, clinical readiness depends not only on accuracy metrics but also on external validation, prospective testing, explainability, regulatory approval, workflow integration, and real-world implementation. At present, only a limited number of systems have been evaluated under real-world clinical conditions.

Economic considerations also play an important role in the implementation of AI-based diagnostic tools. Improved detection accuracy may lead to more efficient utilization of healthcare resources by optimizing the use of ancillary investigations, such as magnetic resonance imaging (MRI), optical coherence tomography (OCT), and visual field (VF) testing. By enabling more accurate triage and reducing unnecessary referrals or imaging, AI-assisted systems have the potential to lower healthcare costs while maintaining or improving diagnostic quality.

### 4.3. Methodological Quality and Risk of Bias

The QUADAS-2 assessment highlights important methodological limitations in the current body of evidence. Although several high-quality studies demonstrated low risk of bias, the majority were affected by moderate or unclear risk, primarily driven by retrospective study designs and limited reporting of patient selection methods.

Some studies [55,61] demonstrated a low risk of bias across key domains, particularly those utilizing deep learning algorithms with external validation. These studies exhibited strong design characteristics, including well-defined reference standards and appropriate patient selection, contributing to the robustness of their findings.

However, some variability in quality was observed. Some studies, such as those by Akbar et al. [62] and Saba et al. [59], were associated with a higher risk of bias, primarily due to limited dataset sizes and lack of external validation. These limitations raise concerns regarding overfitting and reduced generalizability.

Applicability concerns were generally low across the included studies. Nevertheless, studies based on retrospective designs or single-centre datasets may not fully reflect real-world clinical variability. Such limitations highlight the importance of conducting multi-centre, prospective studies to validate AI performance across diverse patient populations.

Importantly, a consistent finding across the review was that deep learning models outperformed traditional machine learning approaches. This superiority was evident not only in terms of diagnostic accuracy but also in clinical relevance, as deep learning models demonstrated greater robustness in handling complex image data.

### 4.4. Comparison with the Existing Literature

The findings of this review are consistent with prior systematic reviews, including those by Anandi et al. [38] and Rambabu et al. [34], which similarly report high diagnostic accuracy of AI systems in ophthalmology. These studies emphasize the advantages of deep learning over traditional machine learning techniques and highlight AI’s potential for deployment in resource-limited settings.

However, the present review provides a more focused evaluation of AI specifically for papilledema detection and grading. In contrast, broader reviews, such as that by Grzybowski et al. [32], examine a wider spectrum of ophthalmic conditions and often include comparisons with non-AI diagnostic modalities, such as optical coherence tomography (OCT) and conventional fundus examination. The systematic review by Li and Wan [45] evaluates a broader spectrum of optic neuropathies, including glaucoma and optic neuritis, rather than focusing specifically on papilledema. Moreover, their analysis incorporates comparisons between artificial intelligence-based approaches and conventional diagnostic modalities, such as optical coherence tomography (OCT) and fundus photography, without AI assistance. In contrast, the present review adopts a more focused scope, specifically examining AI applications in papilledema and emphasizing comparisons between AI systems and expert clinicians. This narrower focus allows for a more targeted assessment of diagnostic accuracy and highlights the potential role of AI as a decision-support tool in neuro-ophthalmologic practice.

Another distinguishing feature of this review is its emphasis on real-world implementation challenges. While earlier reviews primarily focused on diagnostic performance, the current analysis places greater importance on issues such as generalizability, interpretability, and integration into clinical workflows. These factors are critical for translating AI from research settings into routine clinical practice. Our review places strong emphasis on the limitations of AI models, like potential bias in training datasets (e.g., lack of diverse ethnic representation), challenges with external validation and real-world implementation issues, the lack of easy interpretation in deep learning models, leading to probable hesitation from clinicians in adopting AI-driven diagnosis. Other reviews, such as Rambabu et al. [34], insist more on methodological frameworks and little on practical implementation challenges.

### 4.5. Limitations and Challenges

Despite the promising results, several challenges continue to limit the full integration of AI into clinical practice.

#### 4.5.1. Generalizability and Dataset Variability

One of the most significant limitations is dataset variability. While some studies, such as Milea et al. [61] and Lin et al. [57], utilized multiethnic datasets, many models were developed using relatively homogeneous populations. This lack of diversity limits the generalizability of AI systems across different demographic and geographic contexts. Future research must prioritize the inclusion of diverse populations and external validation across multiple centres.

The lack of standardized preprocessing pipelines across studies reflects a broader limitation in AI-based ophthalmic research and may affect the reproducibility and generalizability of model performance.

#### 4.5.2. Real-World Applicability

Another important limitation is the predominance of retrospective study designs. Although these studies provide valuable insights into model performance, they do not fully capture the complexities of real-time clinical environments. Prospective validation studies are needed to assess how AI systems perform in routine practice, including their impact on workflow, clinician behaviour, and patient outcomes.

#### 4.5.3. Interpretability and Trust

The lack of transparency in AI decision-making remains a critical barrier to adoption. Many deep learning models operate as “black boxes” [63], providing outputs without clear explanations. This lack of interpretability may reduce clinician trust and hinder widespread implementation. The development of explainable AI (XAI) is therefore essential to enhance transparency and facilitate clinical acceptance.

#### 4.5.4. Integration and Medico-Legal Considerations

Successful implementation of AI requires seamless integration into existing healthcare systems. This includes compatibility with electronic health record (EHR) systems and alignment with clinical workflows. Additionally, medico-legal concerns related to responsibility and accountability in AI-assisted decision-making remain unresolved [64]. Clear guidelines and legal frameworks will be necessary to address these issues.

#### 4.5.5. Regulatory and Ethical Considerations

Regulatory oversight is another critical factor. The adoption of AI diagnostic tools must comply with established safety and data privacy standards. Agencies, such as the FDA and EMA, will play a central role in defining validation and approval pathways. Ethical considerations, including data security and algorithmic bias, must also be carefully addressed to ensure equitable and safe use of AI technologies [65].

### 4.6. Future Directions

To ensure the clinical utility and scalability of AI in papilledema diagnosis, future research should adopt more rigorous and standardized methodologies.

According to the PICOS framework, future studies should involve large-scale, prospective, multi-centre populations representing diverse ethnic and demographic groups. Such designs will enhance generalizability and provide more reliable estimates of real-world performance.

In addition, the integration of multimodal imaging data—including fundus photography, OCT, and magnetic resonance imaging (MRI)—may further improve diagnostic accuracy. Combining structural and functional data could enable more comprehensive assessment and better differentiation of complex cases.

Future studies should also include direct, real-time comparisons between AI systems and human experts to evaluate performance in clinical settings beyond retrospective analyses. Importantly, outcome measures should extend beyond diagnostic accuracy to include clinical decision-making, therapeutic outcomes, and cost-effectiveness.

Randomized controlled trials and prospective validation studies will be essential to establish the true effectiveness of AI systems. Furthermore, the development of hybrid AI–clinician models, in which AI serves as a decision-support tool rather than a replacement, may represent the most practical and ethically sound approach.

Although CNNs remain the dominant architecture in the studies included in this review, artificial intelligence is evolving rapidly. More recently, Vision Transformers (ViTs) and multimodal deep learning systems have demonstrated promising performance in ophthalmic image analysis by capturing long-range image relationships and integrating information from multiple data sources. While none of the studies meeting our predefined inclusion criteria employed these emerging architectures for papilledema detection, they represent important future directions for research.

The advancement of explainable AI (XAI) is another key priority. Improving interpretability will enhance clinician trust and facilitate broader adoption. The integration of XAI techniques is essential to improve the transparency and clinical integration of deep learning models, particularly in challenging tasks, such as differentiating papilledema from pseudopapilledema.

Additionally, standardized reporting guidelines and validation frameworks should be established to ensure consistency and reproducibility across studies.

## 5. Conclusions

This systematic review provides strong evidence that artificial intelligence, particularly deep learning-based models such as DenseNet, U-Net, and BONSAI, offers high diagnostic accuracy in the detection and grading of papilledema. With sensitivity values ranging from 84.0% to 98% and AUC scores up to 0.99, AI systems have demonstrated performance comparable to, or exceeding, that of expert neuro-ophthalmologists.

Beyond diagnostic accuracy, AI shows significant potential to enhance clinical workflows, improve triage efficiency, and expand access to care, particularly in emergency and resource-limited settings. However, important challenges remain, including issues related to generalizability, interpretability, real-world validation, and integration into clinical practice.

Addressing these limitations through robust study design, regulatory oversight, and technological innovation will be essential for translating AI into routine clinical use. With continued development and validation, AI has the potential to become a reliable, safe, and widely accessible tool in neuro-ophthalmological diagnostics.

## Figures and Tables

**Table 1:** Key characteristics of full-text assesed excluded studies.

No, Reference	Authors	Title	Year	Reason for Exclusion
1 [31]	Branco J, Wang JK, Elze T, Garvin MK, Pasquale LR, Kardon R et al.	Classifying and quantifying changes in papilloedema using machine learning	2024	Focuses on treatment of papilledema rather than diagnostic accuracy, as well as on longitudinal monitoring and quantification of papilledema progression, rather than primary diagnostic detection or classification
2 [32]	Grzybowski A, Jin K, Zhou J, Pan X, Wang M, Ye J et al.	Retina Fundus Photograph-Based Artificial Intelligence Algorithms in Medicine: A Systematic Review	2024	Systematic review; does not present new primary data on papilledema diagnostic accuracy
3 [33]	Nam Y, Kim J, Kim K, Park KA, Kang M, Cho BH et al.	Deep Learning-Based Optic Disc Classification Is Affected by the Presence of Tilted Disc	2024	Focuses on the impact of optic disc tilt on deep learning model performance rather than specifically assessing papilledema
4 [34]	Rambabu L, Smith BG, Tumpa S, Kohler K, Kolias AG, Hutchinson PJ et al.	Artificial intelligence-enabled ophthalmoscopy for papilledema: a systematic review protocol	2024	Systematic review protocol; does not present primary data on papilledema
5 [35]	Salaheldin AM, Abdel Wahed M, Talaat M, and Saleh N	Deep Learning-Based Automated Detection and Grading of Papilledema From OCT Images: A Promising Approach for Improved Clinical Diagnosis and Management	2024	Primarily a model-development study based on OCT imaging, without direct clinical validation using fundus photograph-based papilledema diagnosis
6 [36]	Sathianvichitr K, Najjar RP, Tang Z, Fraser JA, Yau CWL, Girard MJA et al.	A Deep Learning Approach for Accurate Discrimination Between Optic Disc Drusen and Papilledema on Fundus Photographs	2024	Focused primarily on differentiation between papilledema and optic disc drusen (pseudopapilledema) rather than standalone papilledema detection or grading
7 [37]	Yanoff M	Advances in Ophthalmology and Optometry, 2024	2024	Book; general overview
8 [38]	Anandi L, Budihardja BM, Anggraini E, Badjrai RA, and Nusanti S	The use of artificial intelligence in detecting papilledema from fundus photographs	2023	Systematic review; does not present new primary data on papilledema
9 [39]	Chan E, Tang Z, Najjar RP, Narayanaswamy A, Sathianvichitr K, Newman NJ et al.	A Deep Learning System for Automated Quality Evaluation of Optic Disc Photographs in Neuro-Ophthalmic Disorders	2023	Focuses on image quality assessment rather than directly evaluating papilledema
10 [40]	Melissa W. Ko	Tele-Neuro-Ophthalmology	2023	Book chapter; general overview of telemedicine
11 [41]	Sathianvichitr K, Lamoureux O, Nakada S, Tang Z, Schmetterer L, Chen C et al.	Through the Eyes into the Brain, Using Artificial Intelligence	2023	Focuses on multiple neurological abnormalities, not exclusively papilledema
12 [42]	Sun G, Wang X, Xu L, Li C, Wang W, Yi Z et al.	Deep Learning for the Detection of Multiple Fundus Diseases Using Ultra-widefield Images	2023	Focuses on detecting multiple fundus diseases rather than specifically assessing papilledema
13 [43]	Vasseneix C, Nusinovici S, Xu X, Hwang JM, Hamann S, Chen JJ et al.	Deep Learning System Outperforms Clinicians in Identifying Optic Disc Abnormalities	2023	Focused on broad optic disc abnormality classification, with papilledema included as one component rather than the primary outcome
14 [44]	Biousse V, Najjar R, Sathianvichitr K, Tang Z, Hamann S, Fraser C et al.	Deep Learning Can Accurately Distinguish Between True Papilledema and Optic Disc Drusen On Ocular Fundus Photographs	2022	Focused primarily on differentiation between papilledema and optic disc drusen (pseudopapilledema) rather than standalone papilledema detection or grading
15 [45]	Li M and Wan C	The use of deep learning technology for the detection of optic neuropathy	2022	Systematic review; does not present new primary data on papilledema
16 [46]	Wang Z, Keane PA, Chiang M, Cheung CY, Wong TY, and Ting DSW	Artificial Intelligence and Deep Learning in Ophthalmology	2022	Book chapter; general overview, not focused on papilledema diagnostic accuracy
17 [47]	Wang C, Zhang Y, Xu S, Liu Y, Xie L, Wu C et al.	Research on Assistant Diagnosis of Fundus Optic Neuropathy Based on Deep Learning	2022	Focuses on differentiating various optic neuropathies, not exclusively papilledema
18 [48]	Wang JK, Garvin MK, Kupersmith MJ, and Kardon RH	Quantifying Spatial Patterns of OCT Total Retinal Thickness (TRT) in Papilledema Over Time using a Deep Learning Variational AutoEncoder	2022	Focus on OCT imaging technique rather than diagnostic accuracy assessment
19 [49]	Li B, Chen H, Zhang B, Yuan M, Jin X, Lei B et al.	Development and evaluation of a deep learning model for the detection of multiple fundus diseases based on colour fundus photography	2021	Focuses on detecting multiple fundus diseases rather than specifically assessing diagnostic accuracy for papilledema
20 [50]	Biousse V, Newman NJ, Najjar RP, Vasseneix C, Xu X, Ting DSW et al.	Optic Disc Classification by Deep Learning versus Expert Neuro-Ophthalmologists	2020	Focused on multiclass optic disc abnormality classification rather than dedicated papilledema detection
21 [51]	Islam MS, Wang JK, Johnson SS, Thurtell MJ, Kardon RH, and Garvin MK	A Deep-Learning Approach for Automated OCT En-Face Retinal Vessel Segmentation in Cases of Optic Disc Swelling Using Multiple En-Face Images as Input	2020	Focuses on OCT imaging technique development rather than assessing diagnostic accuracy in a clinical context
22 [52]	Islam MS, Wang JK, Deng W, Thurtell MJ, Kardon RH, and Garvin MK	Deep-Learning-Based Estimation of 3D Optic-Nerve-Head Shape from 2D Color Fundus Photographs in Cases of Optic Disc Swelling	2020	Focused on 3D optic nerve head reconstruction and general optic disc swelling analysis rather than direct clinical detection of papilledema
23 [53]	Leong YY, Vasseneix C, Finkelstein MT, Milea D, Najjar R P	Artificial Intelligence Meets Neuro-Ophthalmology	2022	Editorial; does not present original research or primary diagnostic accuracy data
24 [54]	Newman N, Najjar R, Vasseneix C, Zhubo J, Ting D, Liu Y et al.	Human vs. Machine: The Brain and Optic Nerve Study with Artificial Intelligence (BONSAI)	2020	Supplement issue: Conference abstract; lacks full methodological details

**Table 2:** Studies included in the review.

No., Reference	Authors	Title	Journal, Year
1 [55]	Biousse V, Najjar RP, Tang Z, Lin MY, Wright DW, Keahey MT et al.	Application of a Deep Learning System to Detect Papilledema on Nonmydriatic Ocular Fundus Photographs in an Emergency Department	Am J Ophthalmol, 2024
2 [56]	Chang MY, Heidary G, Beres S, Pineles SL, Gaier ED, Gise R et al.	Artificial Intelligence to Differentiate Pediatric Pseudopapilledema and True Papilledema on Fundus Photographs	Ophthalmol. Sci., 2024
3 [57]	Lin MY, Najjar RP, Tang Z, Cioplean D, Dragomir M, Chia A et al.	The BONSAI Deep Learning System for Pediatric Papilledema Detection	J AAPOS, 2024
4 [58]	Azarmina M, Mahmoudi Nejad Azar S, Naghibzadeh SK, Aminzadeh H, Bagheri M et al.	AI Accuracy in Papilledema Diagnosis in Fundus Photographs within 201 Eyes	Biomed J Sci & Tech Res, 2023
5 [59]	Saba T, Akbar S, Kolivand H, and Bahaj SA	Automatic detection of papilledema through fundus retinal images using deep learning	Microsc Res Tech, 2021
6 [60]	Vasseneix C, Najjar RP, Xu X, Tang Z, Loo JL, Singhal S et al.	Accuracy of a Deep Learning System for Classification of Papilledema Severity on Ocular Fundus Photographs	Neurology, 2021
7 [61]	Milea D, Najjar RP, Jiang Z, Ting D, Vasseneix C, Xu X et al.	Artificial Intelligence to Detect Papilledema from Ocular Fundus Photographs	N Engl J Med, 2020
8 [62]	Akbar S, Akram MU, Sharif M, Tariq A, and Yasin UU	Decision Support System for Detection of Papilledema through Fundus Retinal Images	J Med Syst, 2017

**Table 3:** Detailed study characteristics.

Study	Methods	Participants, Aquisitions	Model Type	Centre, Study Type	External Validation, Method	Training/Validation Strategy	Outcomes
Biousse et al. (2024) [55]	AI-assisted triage in emergency department settings	1608 fundus photographs, ED patients, non-mydriatic	BONSAI deep learning system	Single-centre, real-world implementation, prospective	Yes; expert panel	BONSAI model previously trained; prospective real-world validation in emergency department cohort	AUC 0.97; sensitivity 84.0%; specificity 98.9%; improved ED triage efficiency
Chang et al. (2024) [56]	Retrospective image-based classification of papilledema vs. pseudopapilledema using deep learning; 10-fold cross-validation	235 pediatric patients (<18 years), 851 fundus photographs, mydriasis not specified (probable)	DenseNet-based tri-branch CNN	Multi-centre, retrospective clinical study	Yes; separate external test set	10-fold cross-validation with independent external test set	AUC 0.81 (external); sensitivity 90.4%; specificity 56–67%; higher sensitivity than experts, particularly for mild cases
Lin et al. (2024) [57]	Pediatric AI model validated across multiple centres	898 fundus photographs, pediatric patients from three centres, mydriatic	BONSAI deep learning system optimized for pediatric use	Multi-centre, retrospective	Yes; multi-centre external validation	Multi-centre training and external validation across three pediatric cohorts	AUC 0.98; sensitivity 98.0%; specificity 94.1% in pediatric cases
Azarmina et al. (2023) [58]	Retrospective study; AI vs. clinician comparison	201 eyes, adult population, mydriatic	CAD system vs. expert evaluation	Single-centre, retrospective	No; clinician comparison	Retrospective comparison with clinician grading; validation strategy not fully reported	85% agreement with neuro-ophthalmologists
Saba et al. (2021) [59]	AI-based optic disc segmentation and vessel analysis	100 fundus photographs, mydriatic	DenseNet + U-Net segmentation for grading severity	Single-centre, retrospective	No; expert panel	Internal validation on limited dataset; no external validation	Accuracy 99.17%; superior vessel segmentation
Vasseneix et al. (2021) [60]	Deep learning applied for severity grading	2103 fundus photographs, neuroophthalmology clinic, mydriatic	BONSAI deep learning system for severity classification	Single-centre, retrospective	No; expert panel	Internal validation with expert comparison; no external validation	AUC 0.93; effective papilledema severity classification
Milea et al. (2020) [61]	Deep learning validation on large multiethnic dataset	15,846 fundus photographs, multiethnic dataset, mydriatic	Deep learning models (DenseNet, U-Net) for classification	Multi-centre, retrospective	Yes; external testing	Development and external validation on large multiethnic datasets	AUC 0.99; sensitivity 96.4%; specificity 84.7%
Akbar et al. (2017) [62]	Machine learning classification with cross-validation	160 fundus images, hospital-based dataset, mydriatic	SVM classifier using handcrafted papilledema-related image features	Single-centre, retrospective	No; cross-validation	Cross-validation on hospital-based dataset; no external validation	Accuracy 92.86% in classifying papilledema

AI, artificial Intelligence; AUC, Area under the curve; BONSAI, Brain and Optic Nerve Study with Artificial Intelligence; CAD, computer-aided diagnosis; CNN, convolutional neural network; ED, emergency department; SVM, support vector machine.

**Table 4:** QUADAS-2 quality assessment results.

No.	Study	Patient Selection Bias	Index Test Bias	Reference Standard Bias	Flow & Timing Bias	Overall Risk of Bias
1	Biousse et al. (2024) [55]	Low	Low	Low	Low	Low
2	Chang et al. (2024) [56]	Moderate	Low	Low	Moderate	Low
3	Lin et al. (2024) [57]	Moderate	Moderate	Low	Moderate	Moderate
4	Azarmina et al. (2023) [58]	Low	Low	Low	High	Moderate
5	Saba et al. (2021) [59]	High	Moderate	High	High	High
6	Vasseneix et al. (2021) [60]	Moderate	Low	Moderate	Moderate	Moderate
7	Milea et al. (2020) [61]	Low	Low	Low	Low	Low
8	Akbar et al. (2017) [62]	High	Moderate	High	High	High

**Table 5:** Severity assessment of papilledema.

No.	Study	Did It Assess Grading/Severity?	Grading Approach	Key Comment
1	Biousse et al. (2024) [55]	No/limited	Mainly detection of papilledema and optic disc abnormalities	Focused on emergency department detection using non-mydriatic fundus photographs, not formal severity grading.
2	Chang et al. (2024) [56]	Indirectly	Differentiation of true papilledema vs. pseudopapilledema; attention to mild cases	Useful for detecting mild pediatric papilledema, but not primarily a severity-grading study.
3	Lin et al. (2024) [57]	Limited/yes	Pediatric detection; some grading-related analysis	The BONSAI system was mainly evaluated for pediatric papilledema detection, with relevance to grading, but less focused on full severity stratification than Vasseneix et al.
4	Azarmina et al. (2023) [58]	Yes	Frisén score assigned to fundus photographs	Evaluated fundus photographs using Frisén grading and CAD assessment.
5	Saba et al. (2021) [59]	Yes	Mild vs. severe papilledema	Used DenseNet for detection and U-Net-derived vascular indices for grading; reported mild/severe classification.
6	Vasseneix et al. (2021) [60]	Yes	Papilledema severity classification, Frisén-based	The most directly relevant grading study; classified severity on mydriatic fundus photographs and performed comparably to neuro-ophthalmologists.
7	Milea et al. (2020) [61]	No/limited	Multiclass detection: normal, papilledema, other optic disc abnormality	Landmark detection study, but not primarily designed for severity grading.
8	Akbar et al. (2017) [62]	Yes	Mild vs. severe papilledema	Early decision-support system for both detection and grading; reported high accuracy for mild vs. severe classification.

## Data Availability

The original contributions presented in this study are included in the article/Supplementary Material. Further inquiries can be directed to the corresponding author.

## Supplementary Materials

The following supporting information can be downloaded at: https://www.mdpi.com/article/10.3390/jcm15134878/s1, PRISMA Checklist (File S1).

## Abbreviations

The following abbreviations are used in this manuscript:

AI	Artificial Intelligence
AMD	Age-Related Macular Degeneration
ANN	Artificial Neural Network
AUC	Area Under the Curve
BONSAI	Brain and Optic Nerve Study with Artificial Intelligence
CAD	Computer-Aided Diagnosis
CNN	Convolutional neural network
CSF	Cerebrospinal Fluid
DFN	Deep Feedforward Network
DL	Deep Learning
ED	Emergency Department
EHR	Electronic Health Record
EMA	European Medicines Agency
FDA	U.S. Food and Drug Administration
iDx-DR	Named FDA-Cleared Diabetic Retinopathy System
ICP	Intracranial Pressure
IRD	Inherited Retinal Disorder
KNN	K-Nearest Neighbours
MGD	Meibomian Gland Dysfunction
ML	Machine Learning
MLP	Multilayer Perceptron
MRI	Magnetic Resonance Imaging
NN	Neural Network
OCT	Optical Coherence Tomography
PICOS	Population, Intervention, Comparator, Outcomes, Study Design
PRISMA	Preferred Reporting Items for Systematic Reviews and Meta-Analyses
PUN	Pre-Trained Unsupervised Network
QUADAS-2	Quality Assessment of Diagnostic Accuracy Studies, Version 2
RCT	Randomized Controlled Trial
RGB	Red, Green, and Blue
ROP	Retinopathy of Prematurity
SVM	Support Vector Machine
TRT	Total Retinal Thickness
U-Net	Convolutional Neural Network Architecture for Image Segmentation
VF	Visual Field
VGG	Visual Geometry Group
ViT	Vision Transformer
XAI	Explainable Artificial Intelligence
